# Detection of germline CNVs from gene panel data: benchmarking the state of the art

**DOI:** 10.1093/bib/bbae645

**Published:** 2024-12-12

**Authors:** Elisabet Munté, Carla Roca, Jesús Del Valle, Lidia Feliubadaló, Marta Pineda, Bernat Gel, Elisabeth Castellanos, Barbara Rivera, David Cordero, Víctor Moreno, Conxi Lázaro, José Marcos Moreno-Cabrera

**Affiliations:** Hereditary Cancer Program, Catalan Institute of Oncology, Institut d’Investigació Biomèdica de Bellvitge - IDIBELL-ONCOBELL, Avinguda de la Granvia de l’Hospitalet, 199, 08908 L’Hospitalet de Llobregat, Spain; Doctoral Programme in Biomedicine, University of Barcelona (UB), Casanova 143, 08036 Barcelona, Spain; Hereditary Cancer Program, Catalan Institute of Oncology, Institut d’Investigació Biomèdica de Bellvitge - IDIBELL-ONCOBELL, Avinguda de la Granvia de l’Hospitalet, 199, 08908 L’Hospitalet de Llobregat, Spain; Doctoral Programme in Biomedicine, University of Barcelona (UB), Casanova 143, 08036 Barcelona, Spain; Hereditary Cancer Program, Catalan Institute of Oncology, Institut d’Investigació Biomèdica de Bellvitge - IDIBELL-ONCOBELL, Avinguda de la Granvia de l’Hospitalet, 199, 08908 L’Hospitalet de Llobregat, Spain; Centro de Investigación Biomédica en Red de Cáncer (CIBERONC), Instituto de Salud Carlos III, Monforte de Lemos 5, 28029 Madrid, Spain; Hereditary Cancer Program, Catalan Institute of Oncology, Institut d’Investigació Biomèdica de Bellvitge - IDIBELL-ONCOBELL, Avinguda de la Granvia de l’Hospitalet, 199, 08908 L’Hospitalet de Llobregat, Spain; Centro de Investigación Biomédica en Red de Cáncer (CIBERONC), Instituto de Salud Carlos III, Monforte de Lemos 5, 28029 Madrid, Spain; Hereditary Cancer Program, Catalan Institute of Oncology, Institut d’Investigació Biomèdica de Bellvitge - IDIBELL-ONCOBELL, Avinguda de la Granvia de l’Hospitalet, 199, 08908 L’Hospitalet de Llobregat, Spain; Centro de Investigación Biomédica en Red de Cáncer (CIBERONC), Instituto de Salud Carlos III, Monforte de Lemos 5, 28029 Madrid, Spain; Hereditary Cancer Group, Germans Trias i Pujol Research Institute (IGTP), Can Ruti Campus, Camí de les Escoles s/n, 08916 Badalona, Barcelona, Spain; Clinical Genomics Research Group, Germans Trias i Pujol Research Institute (IGTP), Can Ruti Campus, Camí de les Escoles s/n, Badalona, Barcelona, Spain; Genetics Department, Germans Trias i Pujol University Hospital (HUGTiP), Can Ruti Campus, Carretera de Canyet s/n, 08916 Badalona, Barcelona, Spain; Hereditary Cancer Program, Catalan Institute of Oncology, Institut d’Investigació Biomèdica de Bellvitge - IDIBELL-ONCOBELL, Avinguda de la Granvia de l’Hospitalet, 199, 08908 L’Hospitalet de Llobregat, Spain; Lady Davis Institute and Segal Cancer Centre, Jewish General Hospital, 3755 Chemin de la Côte-Sainte-Catherine, Montreal, QC, Canada; Gerald Bronfman Department of Oncology, McGill University, 5100 de Maisonneuve Blvd. West, Suite 720 Montreal, QC, Canada; Unit of Bioinformatics for Precision Oncology (UBOP), Catalan Institute of Oncology, Avinguda de la Granvia de l’Hospitalet, 199, 08908 L'Hospitalet de Llobregat, Barcelona, Spain; Preclinical and Experimental Research in Thoracic Tumors (PReTT), ONCOBELL Program, Institut d’Investigació Biomèdica de Bellvitge (IDIBELL), Avinguda de la Granvia de l’Hospitalet, 199, 08908 L'Hospitalet de Llobregat, Barcelona, Spain; Consorcio de Investigación Biomédica en Red de Epidemiología y Salud Pública (CIBERESP), Instituto de Salud Carlos III, Monforte de Lemos 5, 28029 Madrid, Spain; Consorcio de Investigación Biomédica en Red de Epidemiología y Salud Pública (CIBERESP), Instituto de Salud Carlos III, Monforte de Lemos 5, 28029 Madrid, Spain; Oncology Data Analytics Program (ODAP), Catalan Institute of Oncology, Avinguda de la Granvia de l’Hospitalet, 199, 08908 L'Hospitalet de Llobregat, Barcelona, Spain; Colorectal Cancer Group, ONCOBELL Program, Institut d’Investigació Biomèdica de Bellvitge (IDIBELL), Avinguda de la Granvia de l’Hospitalet, 199, 08908 L'Hospitalet de Llobregat, Barcelona, Spain; Department of Clinical Sciences, Faculty of Medicine and Health Sciences, Universitat de Barcelona Institute of Complex Systems (UBICS), University of Barcelona (UB), Freixa Llarga s/n, 08907 L'Hospitalet de Llobregat, Barcelona, Spain; Hereditary Cancer Program, Catalan Institute of Oncology, Institut d’Investigació Biomèdica de Bellvitge - IDIBELL-ONCOBELL, Avinguda de la Granvia de l’Hospitalet, 199, 08908 L’Hospitalet de Llobregat, Spain; Centro de Investigación Biomédica en Red de Cáncer (CIBERONC), Instituto de Salud Carlos III, Monforte de Lemos 5, 28029 Madrid, Spain; Hereditary Cancer Program, Catalan Institute of Oncology, Institut d’Investigació Biomèdica de Bellvitge - IDIBELL-ONCOBELL, Avinguda de la Granvia de l’Hospitalet, 199, 08908 L’Hospitalet de Llobregat, Spain; Unit of Bioinformatics for Precision Oncology (UBOP), Catalan Institute of Oncology, Avinguda de la Granvia de l’Hospitalet, 199, 08908 L'Hospitalet de Llobregat, Barcelona, Spain; Oncology Data Analytics Program (ODAP), Catalan Institute of Oncology, Avinguda de la Granvia de l’Hospitalet, 199, 08908 L'Hospitalet de Llobregat, Barcelona, Spain

**Keywords:** CNVs, benchmarking, gene panels, germline

## Abstract

Germline copy number variants (CNVs) play a significant role in hereditary diseases. However, the accurate detection of CNVs from targeted next-generation sequencing (NGS) gene panel data remains a challenging task. Several tools for calling CNVs within this context have been published to date, but the available benchmarks suffer from limitations, including testing on simulated data, testing on small datasets, and testing a small subset of published tools. In this work, we conducted a comprehensive benchmarking of 12 tools (Atlas-CNV, ClearCNV, ClinCNV, CNVkit, Cobalt, CODEX2, CoNVaDING, DECoN, ExomeDepth, GATK-gCNV, panelcn.MOPS, VisCap) on four validated gene panel datasets using their default parameters. We also assessed the impact of modifying 107 tool parameters and identified 13 parameter values that we suggest using to improve the tool F1 score. A total of 66 tool pair combinations were also evaluated to produce better meta-callers. Furthermore, we developed CNVbenchmarker2, a framework to help users perform their own evaluations. Our results indicated that in terms of F1 score, ClinCNV and GATK-gCNV were the best CNV callers. Regarding sensitivity, GATK-gCNV also exhibited particularly high performance. The results presented here provide an evaluation of the current state of the art in germline CNV detection from gene panel data and can be used as a reference resource when using any of the tools.

## Graphical Abstract

**Graphical Abstract:**
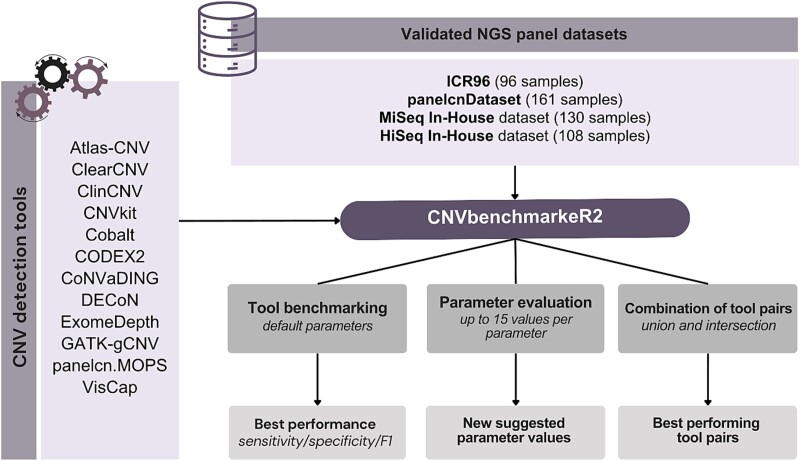

## Introduction

Copy number variants (CNVs) are structural genomic alterations that involve an abnormal number of copies of a DNA segment, resulting in both deletions and duplications. They are a type of structural variation caused by genomic rearrangements, varying in size from 50 bp to several megabases [1, 2]. CNVs are a major source of genomic variation in humans [3]. They affect various biological processes, including evolution, adaptation, and the development and predisposition to diseases such as autism, obesity, and cancer [4–6]. Once a CNV is identified, its clinical significance can be determined, and medical management and prevention measures can be implemented. Their detection is therefore crucial in clinical diagnostics [7].

Several methods to detect CNVs have been developed in recent decades. These methods include Polymerase chain reaction-based methods such as multiplex ligation–dependent probe amplification (MLPA), array-based technologies like microarray-based comparative genomic hybridization (aCGH) or SNP microarrays, massive parallel sequencing, fluorescence *in situ* hybridization (FISH), or Southern blotting. From the above list, MLPA, aCGH, and SNP microarrays are frequently used in diagnostic routines, being MLPA the most common approach for testing one or a few genes [8, 9]. However, these methods are still expensive, time-consuming, and have gene-specific limitations. For example, MLPA relies on single-gene approaches, while aCGH's sensitivity is restricted to sequences within the array's design assembly [2].

The arrival of next-generation sequencing (NGS) has transformed genetic testing by allowing millions of fragments to be sequenced simultaneously [10]. Diagnostic laboratories are using NGS methods to identify multiple types of variation, including CNVs. In diagnostic settings, where laboratories handle a large number of samples, targeted gene panels have emerged as a common and cost-effective approach.

Many bioinformatic tools have been published to identify germline CNVs from NGS data. While most of these tools are reliable for detecting large CNVs, they often struggle to detect small CNVs, especially those spanning a single exon. Furthermore, most tools are not optimized for calling CNVs from targeted gene panel data, as they were originally developed for use with whole-genome or whole-exome data. Beyond addressing these challenges, tools must demonstrate high sensitivity and specificity in diagnostic settings [9]. Therefore, it is crucial to accurately measure tool performance on gene panel data.

Previous studies have evaluated the performance of germline CNV callers on gene panel data. However, these studies suffer from some limitations. Most of them were performed by the tool authors, covered a small subset of currently available tools, and were evaluated on a single dataset [11–15]. To our knowledge, three benchmarks have been published to date by authors who did not evaluate their own tool [16–18]. However, two of them have similar limitations: Roca *et al*. evaluated mainly on simulated data with only a small number of validated CNVs, and Lepkes *et al*. benchmarked four tools on a single dataset where MLPA tests were performed only for CNV calling confirmation. The limitations were partially addressed in our previous work, which evaluated five tools on four real datasets with MLPA results available prior to tool execution [17]. However, our previous work only covered a subset of the tools published until 2018. Moreover, several new tools have been published since 2018, so our previous benchmark provides an incomplete assessment of the current state of the art. Here, we aim to provide a wider, comprehensive, and up-to-date evaluation of germline CNV detection tools on gene panel data by benchmarking 12 tools on four real and publicly available datasets, evaluating the impact of modifying 107 tool parameters and combining tool pairs.

## Material and methods

### Datasets

We defined the criteria for including datasets in the benchmark. These requirements comprised being obtained from gene panel sequencing, having MLPA results before *in silico* calling, including germline single-exon CNVs, and being publicly available. To the best of our knowledge, only four datasets met the requirements as of April 2024, namely, the ICR96 exon CNV validation series (96 samples) [19], a subset of the data used in the panelcn.MOPS publication referred to as panelcnDataset (161 samples) [13], and two in-house datasets (130 and 108 samples sequenced in Illumina MiSeq and HiSeq platforms, respectively) [17]. Table 1 provides further details of the datasets used in this work. All datasets were obtained from hybridization-based capture panels designed for hereditary cancer diagnostics: the TruSight Cancer Panel (Illumina, San Diego, CA) and the ICO-IMPPC Hereditary Cancer Panel (I2HCP) [20]. The bed file defining the regions of interest (ROIs) for the ICR96 and panelcnDataset datasets can be found in Supplementary Table 1, whereas the one for the in-house datasets is in Supplementary Table 2. Datasets contain single and multi-exon CNVs detected in diagnostic routine through MLPA testing. Negative MLPA results, indicating unaffected genes, are also available. MLPA results for each dataset can be found in Supplementary Table 3.

**Table 1:** Datasets used in the benchmark. List of datasets used in the benchmark including the number of samples, validated CNVs (single and multi-exon, deletions, and duplications), sequencing platform employed, availability of the datasets, and additional relevant details. Abbreviations: CNVs, copy number variants; ROI, region of interest; EGA, European Genome-phenome Archive

**Dataset**	**Samples**	**Single-exon CNVs**	**Multi-exon CNVs**	**Deletion CNVs**	**Duplication CNVs**	**Validated genes with CNV**	**Validated ROIS with CNVs**	**Sequencing**	**Availability**	**Additional information**
ICR96	96	25	43	51	17	68	296	TruSight Cancer Panel v2 (100 genes), HiSeq, 2 × 101 bp reads	EGA dataset ID: EGAD00001003335	Samples obtained from one run.
panelcnDataset	161	13	28	36	5	41	321	TruSight Cancer Panel (94 genes), MiSeq, 2 × 151 bp reads	EGA dataset ID: EGAS00001002481	The EGA dataset contains 170 samples, but 9 were excluded for this work (see Supplementary File 1)
In-house MiSeq	130	19	45	56	8	64	394	I2HCP Panel v2.0–v2.2 (122–135 genes), MiSeq, 2 × 300 bp reads	EGA dataset ID: EGAS00001004316	Samples obtained from 48 runs. Three samples had a mosaic CNV.
In-house HiSeq	108	18	40	49	9	58	525	I2HCP panel v2.0–v2.2 (122–135 genes), HiSeq, 2 × 251 bp reads	EGA dataset ID: EGAS00001004316	Samples obtained from 5 runs. Two samples had a mosaic CNV.

Sample alignment was performed using Burrows-Wheeler Aligner (BWA) mem v0.7.17 to the GRCh37 human genome assembly [21, 22] We then used SAMtools v1.16.1 [21] to sort and index Binary Alignment Map (BAM) files and Picard v2.27.4 to include read group information. No further processing or filtering was applied to the BAM files.

### Copy number variant detection tools

The selection of detection tools was based on multiple criteria. Specifically, they must be publicly available, capable of calling germline CNVs at the exon level, designed to work with gene panel data, and not purposely built only for amplicon-based sequencing data. Following the completion of the literature review in December 2023, 12 tools were selected according to these criteria (Table 2): clearCNV v0.306 [23], GATK-gCNV v4.5.0 [24], Atlas-CNV v.0 [15], Cobalt v0.8.0 [25], ClinCNV v1.18.3 [26], CNVkit v0.9.10 [27], VisCap v0.8 [28], DeCoN v2.0.1 [29], panelcn.MOPS v1.20.0 [13], ExomeDepth v1.1.16 [30], CoNVaDING v1.2.1 [11], and CODEX2 v1.3.0 [31]. The latter five were evaluated in our previous work, but we included them here to facilitate tool comparison and to evaluate the most updated versions. Nine germline CNV detection tools were considered for inclusion in this work but were later discarded for multiple reasons: SeqCNV, CNVPanelizer, CNV-Z, ifCNV, SavvyCNV, CCR-CNV, Hadoop-CNV-RF, PattRec, and the pipeline used by Singh *et al*. [9] Supplementary Table 4 provides a list of discarded callers and the reason for their exclusion.

**Table 2:** Benchmarked tools. List of benchmarked tools including version used in the benchmark, programming language, URL for accessing the tool’s code, a brief summary of the CNV detection method, number of parameters examined in the parameter evaluation section, year of publication, number of citations, PubMed ID (PMID), and whether the tool was benchmarked in a previous work of Moreno-Cabrera *et al*. [17].

**Tool**	**Version**	**Language**	**Availability**	**Methods**	**Number of evaluated parameters**	**Year** (paper publication)	**Citations** ^a^	**PMID**	**Benchmarked in** [14]
Atlas-CNV	0	R and Perl program	https://github.com/theodorc/Atlas-CNV	It normalizes individual read depth data to average read depth per target, converting it to reads per kilobase million (RPKM). It computes log2 scores for each sample/median ratio at every exon, assessing sample quality via SampleQC, checking StDev of log2 scores and analysis of variance (ANOVA) on mean RPKM coverage.	2	2019	14	30890783	No
ClearCNV	0.306	Python program	https://github.com/bihealth/clear-cnv	It utilizes match scores to group samples based on coverage patterns. It employs data normalization, scaled *z*-scores, and *r*-scores to identify copy number variations (CNVs) in both multi-exon and single-exon regions.	7	2022	1	35751599	No
ClinCNV	1.18.3	R, Java, Python program	https://github.com/imgag/ClinCNV	ClinCNV employs an algorithm that combines the strengths of circular binary segmentation and hidden Markov model–based techniques to perform multi-sample normalization and CNV calling.	2	2022^b^	6	–	No
CNVkit	0.9.10	Python program	https://github.com/etal/cnvkit	It uses targeted and the nonspecifically captured off-target reads to calculate log2 copy ratios across the genome.	18	2016	1212	27100738	No
Cobalt	0.8.0	Python program	https://github.com/ARUP-NGS/cobalt	It introduces two algorithmic adaptations to improve accuracy in a hidden Markov model. A method for computing target and copy number–specific emission distributions and they perform pointwise maximum *a posteriori* HMM decoding to improve sensitivity for small CNV.	8	2022	0	35854218	No
CODEX2	1.3.0	R package	https://github.com/yuchaojiang/CODEX2	Based on CODEX package, it models the GC content bias and normalizes the read depth data for CNV detection via a Poisson latent factor model.	8	2018	39	30477554	Yes (v.1.2.0)
CoNVaDING	1.2.1	Perl program	https://github.com/molgenis/CoNVaDING	Combination of ratio scores and *Z*-scores of the sample of interest compared to the selected normalized control samples.	7	2016	67	26864275	Yes (v.1.2.0)
DECoN	2.0.1	R program	https://github.com/RahmanTeam/DECoN	Modifies ExomeDepth package by altering the hidden Markov model probabilities to depend upon the distance between exons.	3	2016	59	28459104	Yes (v.1.0.1)
ExomeDepth	1.1.16	R package	https://github.com/vplagnol/ExomeDepth	Beta-binomial model with GC correction and hidden Markov model to combine likelihood across exons.	3	2012	516	22942019	Yes (v.1.1.10)
GATK-gCNV	4.5.0	Java, Python, R program	https://github.com/broadinstitute/gatk	It calculates read counts over specified genomic regions per sample; it clusters technically similar samples using principal component analysis to reduce biases and enhance efficiency. After estimating chromosomal ploidy, it denoises read depth, infers CNVs via a unified model using the Viterbi algorithm	35	2023	0	37604963	No
panelcn.MOPS	1.20.0	R package	https://github.com/bioinf-jku/panelcn.mops	Adaptation of cn.MOPS package, which decomposes variations in coverage across samples into integer copy numbers and noise by means of its mixture components and Poisson distributions.	13	2017	53	28449315	Yes (v.1.0.0)
VisCap	0.8	R program	https://github.com/pughlab/VisCap	It determines the portion of sequence coverage allocated to genomic intervals and calculates log2 ratios compared to the median of reference samples with a matching test setup. CNV candidates are identified when log2 ratios surpass thresholds set by the user.	2	2016	49	26681316	No

^a^Citations obtained on Nov 2023.

^b^Preprint.

### Benchmark evaluation metrics

The performance of each tool was evaluated at two levels: per ROI and per gene. Detailed definitions of both levels can be found in Supplementary File 1.

For each tool, across all dataset and evaluation levels, a range of performance metrics were computed, including sensitivity, specificity, positive predictive value, negative predictive value, false negative rate, false positive rate, F1 score, accuracy, Matthews correlation coefficient, and Cohen’s kappa coefficient (Supplementary File 1). We also measured tool run times on the ICR96 dataset using a workstation with 24 GB random access memory (RAM) and 1 central processing unit (CPU) per job.

### Benchmark execution

We implemented CNVbenchmarkeR2, an R framework that enables the automatic and flexible benchmarking of CNV callers. Code and documentation are available at https://github.com/jpuntomarcos/CNVbenchmarkeR2, so other users can benefit from it to benchmark the tools against their own datasets. We used CNVbenchmarkeR2 to run each tool on each dataset using the default parameters specified in the tool documentation.

The CNVbenchmarkeR2 code shows the steps performed to run each tool. In the case of GATK-gCNV, we followed the guide published by GATK (https://gatk.broadinstitute.org/hc/en-us/articles/360035531152--How-to-Call-rare-germline-copy-number-variants) to call rare germline variants, including the AnnotateIntervals and FilterIntervals steps, both recommended in the guide. However, since these steps are described as optional in the guide and users may ignore their impact on performance, we benchmarked two additional workflows to compare them with the final one. Thus, we benchmarked: (i) the complete workflow (GATK-gCNV) including both AnnotateIntervals and FilterIntervals steps, (ii) a workflow excluding AnnotateIntervals and FilterIntervals steps (GATK-gCNV_no_AI_FI), and (iii) a workflow excluding the AnnotateIntervals step (GATK-gCNV_no_AI), which is the default approach in the GATK germline cohort workflow description language pipeline.

### Parameter evaluation

All tools evaluated in this benchmark have adjustable parameters. However, in most cases, neither the tool documentation nor any other source is clear about the impact on tool performance when these parameters are changed. To address this issue, we systematically evaluated tool parameters by testing them over a wide range of values on all datasets. For numerical parameters, we tested 15 parameter values, including the default one. For categorical parameters, we tested all available options in the tool. We computed the metrics described in the Benchmark Evaluation Metrics section for all executions.

For numerical parameters, we also obtained the optimal range as follows: (i) for each dataset, we identified the parameter value that maximized the F1 score at the ROI level; (ii) The optimal range is defined by the lowest and highest parameter values obtained from the previous step. We used the optimal range to identify parameters where the optimal range is completely below or above the default parameter value. For these parameters, we also determined the suggested parameter value to use, which we defined as the value of the optimal range that is closest to the default value.

### Combination of tool pairs

We assessed the impact of tool pair unions and intersections on performance and ascertained whether any pair was capable of detecting all CNVs. All 66 possible tool pairs were evaluated by combining the results obtained separately when using the default parameters on the four datasets included in this work. Both per ROI and per gene metrics were generated. The R package GenomicRanges v1.48.0 was used to calculate the union and intersection of tool calls.

## Results

### Benchmark with default parameters

The tools were run on every dataset using default parameters. Evaluation metrics were then calculated at two levels: per ROI and per gene (see Material and Methods section for details).

**Figure 1:**
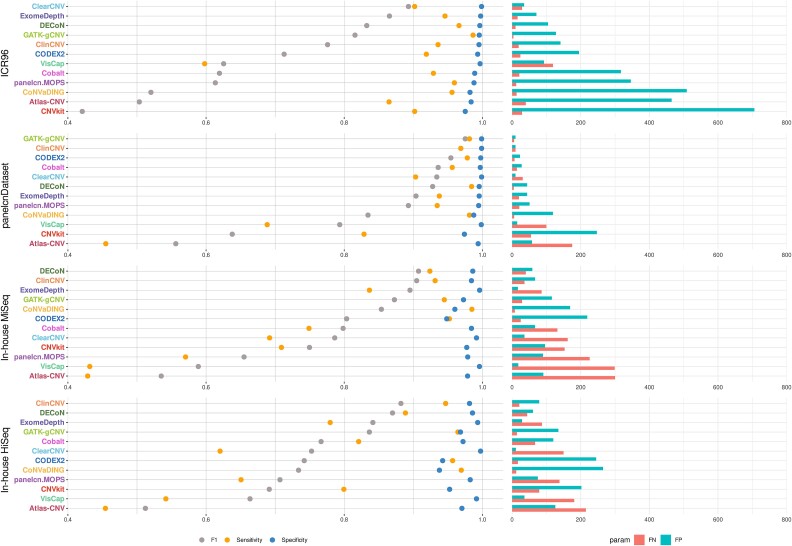
Benchmark results at the ROI level. The tools were run using default parameters and are listed in descending order based on their F1 score in each dataset. (FN, false negative; FP, false positive; F1, F1 score).

Per ROI metrics allow us to assess tool performance at single-exon resolution (Fig. 1, Supplementary Table 5). Regarding the F1 score, a common measure of binary classifier accuracy, tool performance varied widely across datasets, ranging from 0.42 (CNVkit in ICR96) to 0.98 (GATK-gCNV in panelcnDataset). Interestingly, GATK-gCNV and ClinCNV were the only tools to consistently score in the top five for each dataset, with values between 0.78 and 0.98. On the other hand, all tools were highly specific, achieving values over 0.94 in all tool–dataset runs. In terms of sensitivity, we observed more variability, with tools ranging from 0.43 to 0.99. For both the ICR96 and panelcnDataset datasets, all tools except Atlas-CNV and VisCap achieved a sensitivity >0.90. CNVkit exceeded this threshold as well, but only for the ICR96 dataset. However, in the in-house datasets, only ClinCNV, CODEX2, GATK-gCNV, and CoNVaDING achieved sensitivity values >0.90. Supplementary Figure 1 shows per ROI results sorted by sensitivity to facilitate the analysis.

**Figure 2:**
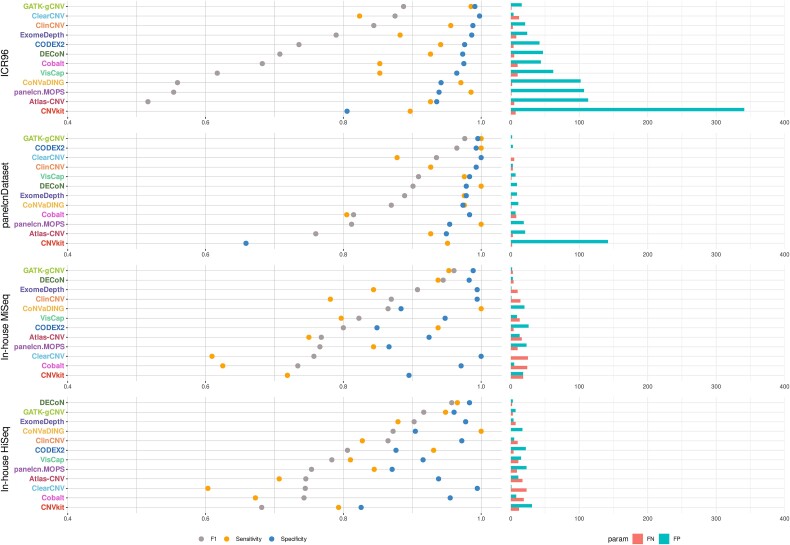
Benchmark results at the gene level. The tools were run using default parameters and are listed in descending order based on their F1 score in each dataset. The F1 scores for CNVkit on ICR96 and panelcnDataset, which were not included in the presented figure, are 0.26 and 0.35, respectively. (FN, false negative; FP, false positive; F1, F1 score).

Figure 2 and Supplementary Table 5 show benchmark results at the gene level, which are particularly relevant in diagnostic settings. Regarding sensitivity, a metric commonly used in diagnostics to assess the classifier’s ability to detect positives, some tools demonstrated high performance. In particular, GATK, CoNVaDING, DECoN, and CODEX2 obtained values >0.93 for each dataset. CoNVaDING showed very high performance in detecting positives: it missed only 3 out of 231 positives across all datasets. GATK-gCNV, DECoN, and CODEX2 missed more positives in total (7, 11, and 12, respectively) but generated fewer false positives (FPs; 27, 62, and 93, respectively) compared to CoNVaDING (150 FPs). On the other hand, ClearCNV and Cobalt were the callers that missed most of the positives: 65 and 61, respectively. Supplementary Figure 2 shows the per gene results sorted by sensitivity. In terms of F1 score, tools showed again large differences with values ranging from 0.26 to 0.98. GATK-gCNV exhibited the highest performance based on this metric: only DECoN surpassed it in the InHouse HiSeq dataset.

Supplementary File 1 shows tool run times obtained in a workstation with 24 GB RAM and 1 CPU per job. The benchmarked tools required a median of 53 minutes to perform CNV calling on the ICR96 dataset. ClinCNV and CODEX2 were the fastest tools, completing the task in 13 and 14 minutes, respectively. In contrast, Atlas-CNV, CNVkit and VisCap were the most time-consuming tools, requiring 147, 148 and 192 minutes, respectively.

### GATK-gCNV workflows

The GATK-gCNV results presented in this work were obtained including the AnnotateIntervals and FilterIntervals steps. However, to better understand the effect of including these steps, two additional workflows were benchmarked (see Methods). Supplementary File 1 shows that certain metrics exhibited considerable variability across workflows. With regard to per ROI metrics, the workflows including the FilterIntervals step demonstrated higher sensitivity across all datasets in comparison to the GATK-gCNV_no_AI_FI workflow, with increases ranging from 0.01 to 0.09. The enhancement was even more pronounced in per gene metrics. In particular, the workflows including the FilterIntervals step demonstrated superior performance compared to GATK-gCNV_no_AI_FI, with gains ranging from 0.03 to 0.30. Furthermore, these two workflows exhibited notable F1 score improvement at the gene level, with values increasing between 0.01 and 0.22.

When comparing both workflows that include the FilterInterval step, GATK-gCNV and GATK-gCNV_no_AI, neither tool demonstrated a clear advantage in terms of sensitivity, specificity, or F1 score across all datasets.

### Parameter evaluation

We systematically varied each tool parameter over a broad range of values to assess its impact on tool performance (see Material and Methods). A total of 6110 executions were conducted to evaluate 107 tool parameters across all datasets. All results are presented in Supplementary Table 6. Additionally, 436 figures containing sensitivity, specificity, and F1 score at the ROI and gene level were also generated and are available at https://doi.org/10.6084/m9.figshare.25930960. Tool users can utilize these results as a guide to understand the expected effect when modifying each parameter.

Modifying each parameter had a different effect on tool performance. We identified four main patterns of performance change: (i) no discernible effect on the tool performance, resulting in flat curves (e.g. DECoN mincorr parameter); (ii) Increase in sensitivity and decrease in specificity or vice versa (e.g. CODEX2 cn_del_threshold parameter); (iii) sensitivity or specificity exhibiting a bell-shaped behavior (e.g. ClearCNV zscale parameter); (iv) performance changes without a distinct pattern, often showing successive increases and decreases in sensitivity (e.g. ClearCNV sample_score_factor parameter).

At the ROI level, we also determined the optimal range for each numerical parameter and identified 13 parameters where the optimal range was completely below or above the default parameter value (Table 3). In such cases, adjusting the default parameter value in one direction, increasing or decreasing it, results in a higher F1 score at the ROI level across all datasets. We therefore identified the suggested parameter value to use as the one within the optimal range closest to the default value.

**Table 3:** Suggested parameter values. Suggested parameter values to be used for improving the F1 score at the ROI level. The suggested value is the value within the optimal range closest to the default value. The mean F1 increase is calculated as the difference between the mean of the F1 scores obtained across datasets using the default value and the mean of the F1 scores obtained across datasets using the suggested value.

**Tool**	**Parameter**	**Default value**	**Optimal range**	**Suggested value**	**Mean F1 increase**
Atlas-CNV	threshold_dup	0.4	[0.44–0.6]	0.44	0.0040 (+0.76%)
ClearCNV	trans_prob	0.001	[0.0015–0.02]	0.0015	0.0006 (+0.07%)
ClinCNV	scoreG	20	[25–50]	25	0.0126 (+1.43%)
CNVkit	alpha (segmetrics)	0.05	[0.0001–0.04]	0.04	0.0006 (+0.09%)
CNVkit	drop-outliers	10	[1–4]	4	0.0080 (+1.26%)
Cobalt	high-depth-trim-frac	0.01	[0.025–0.1]	0.025	0.0033 (+0.50%)
Cobalt	var-cutoff	0.9	[0.91–0.99]	0.91	0.0053 (+0.81%)
CODEX2	cn_del_threshold	1.7	[1.3–1.67]	1.67	0.0172 (+2.14%)
CODEX2	cn_dup_threshold	2.3	[2.5–2.8]	2.5	0.0352 (+4.38%)
CODEX2	gc_thresh_down	20	[30–40]	30	0.0018 (+0.22%)
CoNVaDING	ratioCutOffLow	0.65	[0.5–0.6]	0.6	0.0168 (+2.28%)
panelcn.MOPS	CN3	1.46	[1.6–1.7]	1.6	0.0173 (+2,41%)
panelcn.MOPS	corrThresh	0.99	[0.5–0.985]	0.985	0.0017 (+0,24%)

### Combination of tool pairs

We evaluated all 66 combinations of tool pairs using their parameters set to default (Supplementary Table 7). The union of calls from tool pairs resulted in better sensitivity albeit at the expense of a lower specificity. From an ROI-level perspective, no combination of tools achieved a sensitivity of 1. As per gene level results, five tool pairs achieved the maximum sensitivity across all datasets (Supplementary File 1): Atlas-CNV/CoNVaDING, CODEX2/CoNVaDING, CNVkit/CoNVaDING, panelcn.MOPS/CoNVaDING, and DECoN/CODEX2. Among these pairs, the union of CNVkit and CoNVaDING yielded the lowest specificity in most datasets, with values between 0.65 and 0.84. The other pairs did not show large differences between them and achieved values ranging from 0.78 to 0.97 across datasets.

In contrast, intersecting tool calls increased specificity at the expense of a lower sensitivity. No tool pair achieved perfect specificity across all datasets. However, the intersection of CODEX2/GATK-gCNV and CODEX2/DECoN identified all true CNVs in the panelcnDataset dataset at the gene level, without generating FPs.

## Discussion

The published benchmarks of germline CNV callers for gene panel data suffer from certain limitations. These limitations include evaluating mainly simulated data, evaluating small datasets, or testing only a small subset of published tools [16–18]. Here, we conducted a comprehensive benchmark of 12 tools on four publicly available datasets, using tool default parameters, assessing the impact of changing tool parameter values, and evaluating the combination of tool pairs to produce better meta-callers.

### Benchmark with default parameters

Several approaches can be used to measure tool performance. We have benchmarked tools using two levels of resolution, ROI and gene level, and several metrics such as F1 score, sensitivity, or specificity. These approaches facilitate the analysis of which tools are more suitable in each context. If we focus on the overall performance of the tool, the F1 score is a common metric used to evaluate the performance of binary classifiers. Based on this metric, we highlight GATK-gCNV and ClinCNV, which showed outstanding performance at the ROI level, with GATK-gCNV demonstrating superior performance at the gene level. Therefore, we suggest using ClinCNV and GATK-gCNV when the priority is to maximize the overall performance according to the F1 score and especially the latter when the focus is on the gene level. It is noteworthy that ClinCNV was also the fastest tool, requiring only 13 min to call CNVs. While run time is not typically the primary factor in selecting a calling tool, it may be advantageous in settings where computational resources are limited or results must be delivered as quickly as possible.

On the other hand, sensitivity is a key metric in diagnostic settings, where the aim is usually to minimize the number of FNs. Also, in genetic diagnostics it is frequently useful to focus on the gene level because, if at least one exon from the CNV is detected, a subsequent MLPA test could be performed to confirm the CNV [17]. Focusing on the sensitivity and the per gene metrics, CoNVaDING, GATK-gCNV, CODEX2, and DECoN were among the best five in all datasets. Although we highlight the power of CoNVaDING to detect positives, with only three FNs across all datasets, it also produced a high number of FPs. In contrast, GATK-gCNV demonstrated high sensitivity and high specificity at the same time, which makes it a valuable candidate for use in genetic diagnostics. In any case, the aforementioned tools produced a relevant number of FPs. Since most diagnostic units validate CNV calls using orthogonal methods, the number of FPs should be taken into consideration to ensure the cost-effectiveness of diagnostic routines. On the opposite side, the highest rates of FNs were obtained by ClearCNV and Cobalt. This suggests that, when their default parameters are used, these tools may not be appropriate solutions for calling CNVs in genetic diagnostic settings from NGS panel data.

No previous work has evaluated the sensitivity and specificity of the two most highlighted tools discussed here, GATK-gCNV and ClinCNV, on gene panel data. Demidov *et al.* evaluated the performance of ClinCNV on WGS and WES data in their ClinCNV publication and only compared it with ExomeDepth and DELLY [26]. In the GATK-gCNV publication, the authors demonstrated that GATK-gCNV was capable of achieving 95% sensitivity in detecting CNVs of two or more exons [24]. However, GATK-gCNV was run on WES data, and the methodology differed from that used in the work presented here. Lepkes *et al.* included GATK-gCNV in their benchmark on gene panel data, but MLPA tests were performed after the benchmark execution, preventing the calculation of sensitivity and specificity [16].

### GATK-gCNV workflows

The GATK guide to call rare germline CNVs includes two optional steps: AnnotateIntervals and FilterIntervals. To gain a deeper understanding of the effect of including them, we evaluated alternative GATK-gCNV workflows as detailed in Materials and Methods. Interestingly, the inclusion of the FilterIntervals step had a relevant impact on the performance: the workflows incorporating the FilterIntervals step clearly outperformed the one that excluded it. We consequently recommend including this step for the detection of rare germline CNVs from gene panel data. The GATK guide describes this step as optional but recommended, which may lead some users to overlook this step despite its impact on performance. We believe that the results observed here will encourage users to include the FilterIntervals step in their workflows. On the other hand, the inclusion of the AnnotateIntervals step, which entails explicit guanine and cytosine (GC)-content-based filtering, did not result in a discernible improvement in performance across all datasets. The GATK guide also described this step as optional but recommended, so we suggest users to further validate its effect on their own datasets.

### Parameter evaluation

The available documentation on the effect of each parameter on tool performance is often scarce or nonexistent. Deciding which parameter to modify and how to tune it may be particularly challenging when a tool provides dozens of parameters, as is the case with GATK-gCNV. To address these issues, we repeated benchmark executions, modifying each parameter individually over a range of values. We generated 436 figures that can be used as reference guidance for research and diagnostic laboratories that are currently using or planning to use any of the tools in their settings. These results should help users to better understand the contribution of each parameter on tool performance, prevent them from inadvertently overlooking the most relevant parameters, and facilitate fine-tuning of tool parameters.

Parameters affected performance in multiple ways, and we grouped these changes into four main patterns. Some parameters had no effect on performance and should not be considered when trying to enhance tool performance. Other parameters resulted in the typical trade-off of binary classifiers: an increase in sensitivity leads to a decrease in specificity or vice versa. We recommend modifying this type of parameters to adjust the balance between sensitivity and specificity. Other parameters showed a bell-shaped behavior in sensitivity or specificity, suggesting that the tool performance could be optimized around a certain value. Finally, other parameters affected tool performance without a clear pattern, often with successive increases and decreases in sensitivity. We suggest modifying these parameters with caution as the observed variability makes it difficult to predict their impact on performance.

It is noteworthy that for certain parameters, the highest F1 scores across all datasets were observed on one side of the default parameter, either below or above it. Thus, we were able to identify the optimal range for 13 parameters, wherein the tools demonstrated superior performance compared to the default parameters. One possible explanation for this finding is that the tools were developed for specific datasets, which may restrict their applicability to other datasets, such as those used in this manuscript. Also, the authors may have optimized their tools based on performance metrics other than the F1 score. Anyway, while any value within this range could potentially improve the F1 score, the parameter value we suggested was the closest to the default value. Since it is the closest to the value set by the authors, we understand that this is the most conservative approach.

Using the suggested parameter values resulted in different F1 score increases. Although some yielded modest F1 score increments, such as the trans_prob and alpha parameters, others produced notable F1 score changes. Modifying the cn_dup_threshold, CN3, ratioCutOffLow, and cn_del_threshold parameters resulted in an average F1 increase of >2%. In any case, all suggested parameter values represent an opportunity to enhance the overall performance of the tools, and we recommend tool users to try them on their own datasets for further validation.

### Combination of tool pairs

A common approach in bioinformatics is to join or intersect the results obtained by variant callers separately to produce new meta-callers [32–34]. In this work, no tool was capable of detecting all CNVs at the ROI or gene level with their default parameters. We therefore assessed the effect of tool unions and intersections on performance to determine if any meta-caller could achieve 100% sensitivity.

Although no union of tools detected all true positive ROIs in the per ROI results, five tool pairs did so at the gene level. These pairs may be employed in diagnostic scenarios where no true CNV should be overlooked. Indeed, if a CNV caller or meta-caller is capable of detecting all CNVs, it can be used as a screening step prior to an orthogonal method validation, such as MLPA [35]. This approach has the potential to enhance the mutation detection yield and reduce costs in genetic testing for hereditary cancer [17]. In any case, we observed that the five tool pairs obtained largely different specificity values across the datasets. We hence encourage diagnostic units to conduct a thorough evaluation of their performance on their own in-house datasets.

### Limitations

The results presented in this work have some limitations to note. First, the datasets used have an unusually high frequency of rare CNVs compared to the general population, where CNV frequency is expected to be considerably lower [36]. It would be of interest to assess the performance of the tools on datasets that would more accurately reflect the incidence of CNVs in the general population. Anyway, the datasets used in this benchmark provide a challenging scenario for the evaluation of the tools. Second, regarding the combination of tool pairs and the identification of suggested parameter values, we did not divide the datasets into training and validation subsets to assess whether the tool performance observed in a training subset was confirmed in a validation dataset. Hence, we recommend users to evaluate how tool pairs and suggested parameter values behave on their own datasets. Finally, we only evaluated combinations of tool pairs, leaving open the question of whether a combination of three or more tools could lead to a better meta-caller.

## Conclusion

Here, we conducted a comprehensive evaluation of the current state of the art in germline CNV detection from gene panel data. Although the identification of CNVs remains challenging, our results indicate that certain tools can achieve very high performance. In terms of F1 score, ClinCNV and GATK-gCNV demonstrated superior calling performance compared to the other tools, with GATK-gCNV exhibiting high effectiveness in identifying true positives. The benchmark results, parameter evaluation, combination of tool pairs, and the CNVbenchmarkeR2 framework that we developed can serve as a valuable guide to research and diagnostic teams facing the task of detecting germline CNVs from gene panel data.

**Key Points**

- Comprehensive evaluation of 12 copy number variation callers on four real-validated datasets.
- ClinCNV and GATK-gCNV excelled, with GATK-gCNV achieving superior sensitivity.
- Assessment of the effect of modifying 107 tool parameters: 436 figures are publicly available.
- CNVbenchmarker2 enables users to conduct their own tool evaluations.

## Supplementary Material

Supplementary_data_bbae645

## Data Availability

The datasets underlying this article are available in the European Genome-Phenome Archive (EGA) and can be accessed with the following accession numbers: EGAD00001003335 for ICR96, EGAS00001002481 for panelcnDataset, and EGAS00001004316 for the In-house MiSeq / HiSeq datasets. The CNVbenchmarkeR2 code is publicly available at https://github.com/jpuntomarcos/CNVbenchmarkeR2. Similarly, the complete set of 436 parameter evaluation figures is available for download at https://doi.org/10.6084/m9.figshare.25930960.
