# Supplementary material for: Detection of germline CNVs from gene panel data: benchmarking the state of the art
Source: Brief Bioinform. 2024 Dec 12;26(1):bbae645. doi: 10.1093/bib/bbae645 (PMC11637760; doi:10.1093/bib/bbae645)
Supplement: Supplementary_data_bbae645 [file supplementary_data_bbae645.zip › Supplementary File 1.pdf]

Benchmark evaluation metrics

At the per ROI level, individual ROIs were assessed as standalone entities. They were categorized with correctness labels: True Positive (TP) or True Negative (TN) when the tool outcome was consistent with the MLPA result, False Negative (FN) when the tool missed a CNV identified by MLPA, and False Positive (FP) when the tool erroneously reported a CNV not detected by MLPA. This level of evaluation provides the most detailed assessment.

At the per gene level, since most MLPA kits cover entire genes, genuine CNVs would be confirmed by MLPA when any CNV call was verified within any ROI of the affected gene. Therefore, the per gene metrics assigned a correctness label to each gene, encompassing all its exons: TP if at least one of its ROIs proved to be a TP; FN if MLPA detected a CNV in at least one ROI and none were identified by the tool; FP if the tool indicated a CNV in at least one ROI and none were confirmed by MLPA; TN if neither MLPA nor the tool detected a CNV within any of its ROIs.

Multiple performance metrics were computed for each tool-dataset execution. Sensitivity was defined as TP/(TP + FN), specificity was defined as TN/(TN + FP), positive predictive value (PPV) was defined as TP/(TP + FP), negative predictive value (NPV) was defined as TN/(TN + FN), false negative rate (FNR) was defined as FN/(FN + TP), false positive rate (FPR) was defined as FP/(FP + TN), F1 score (F1) was defined as 2TP/(2TP + FP + FN), accuracy was defined as (TP + TN) / (TP + FN + FP + TN), Matthews correlation coefficient (MCC) was defined as √(sensitivity × specificity × PPV × NPV) - √(FNR × FPR × (1 - NPV) × FDR) and Cohen's kappa coefficient was defined as (2 x (TP x TN – FN x FP)) / ((TP + FP) x (FP + TN) x (TP + FN) x (FN + TN)).

Tool and dataset selection

We followed two approaches for selecting tools for our analysis. First, we conducted searches on Google Scholar, using combinations of keywords including (CNV | CNA | copy number variants | copy number alterations) & (panel data | targeted panels | targeted gene panels) & germline. In addition to this search, we reviewed several articles that either cited benchmark or reviews for CNVs or referenced other CNV tools specifically used in panel data applications.

Dataset selection was performed through 1) review of the data used in germline CNV caller manuscripts, 2) search through Google Scholar and 3) search in the European Genome-phenome Archive (EGA) repository. Only four datasets met the criteria to be included in this manuscript.

Samples used in dataset panelcnDataset

The dataset EGAS00001002481 contains 170 samples. However, 9 samples were removed to form the final dataset panelcnDataset used in this benchmark. The excluded samples showed alterations out of the scope of this benchmark: 5 presented CNVs smaller than an exon (IBK9, IBK23, IBK67, IBK153, IBK166) and 4 contained ALUs insertions instead of CNVs (IBK141, IBK142, IBK143, IBK151). IBK141 ALU insertion was identified in our previous work (*Moreno-Cabrera et al.*, 2020).

Bed files generation

For TruSight-based datasets, ICR96 and panelcnDataset, we employed a modified version of the target bed file previously published (Fowler et al. 2016). The adjustments included the removal of the fourth column, addition of a gene column, join of overlapping regions, and ultimately sorting the bed file by chromosome and start position. For in-house datasets, we generated a target bed file encompassing all coding exons derived from protein-coding transcripts within the I2HCP panel. These genomic coordinates were extracted from Ensembl BioMart version 108.

ClinCNV used an annotated version of the bed files which included a GC content column. This bed files were generated using the BedAnnotateGC tool.

DECoN installation

For the execution of DECoN, we modified the package environment's configuration due to compatibility issues encountered with ExomeDepth version 1.1.15. Specifically, we updated the renv.lock file to specify the use of ExomeDepth version 1.1.16. This adjustment addresses the problem reported in issue #45 on the DECoN GitHub repository (https://github.com/RahmanTeam/DECoN/issues/45).

CODEX2 code adaptation

The CODEX2 parameters were obtained from the CODEX2 targeted demo that the authors published. In order to further parameterize CODEX2, we also added the cn_del_threshold and cn_dup_threshold parameters, whose default values in the original demo were 1.7 and 2.3, respectively.

Run-times

We measured tool run-times on the ICR96 dataset using a workstation with 24 GB RAM and 1 CPU per job.

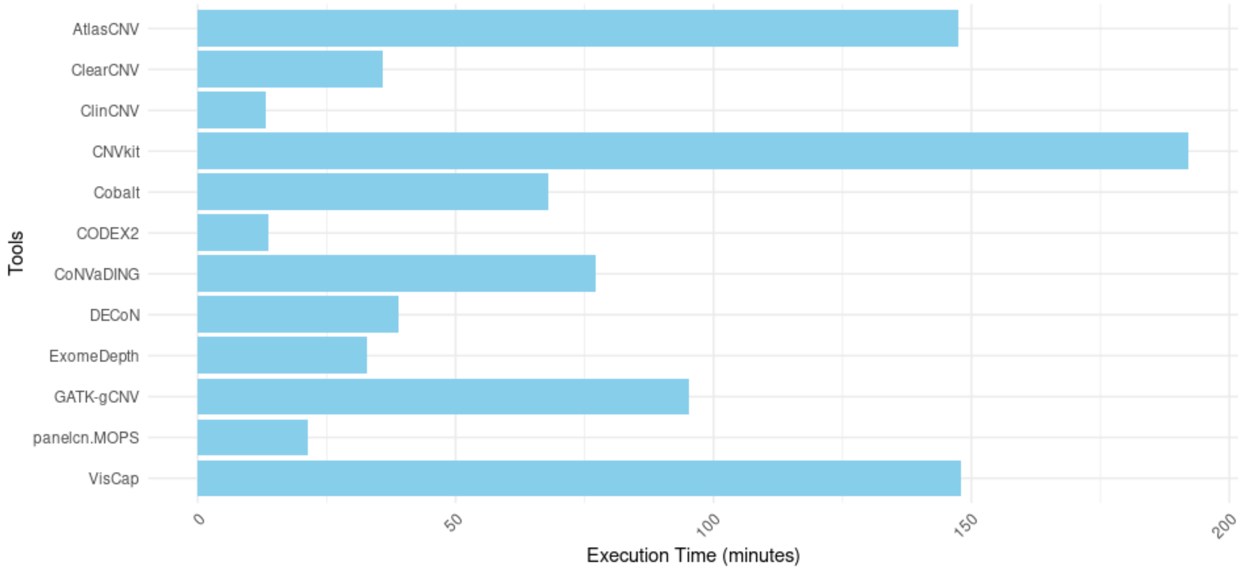

Tool run-times (minutes) using 24GB of memory and 1 core. The data was collected employing the default parameters on the ICR96 dataset. Values: Atlas-CNV 147,47; ClearCNV 35,92; ClinCNV 13,15; CNVkit 192,05; Cobalt 68,02; CODEX2 13,73; CoNVaDING 77,03; DECoN 38,93; ExomeDepth 32,72; GATK-gCNV 95,17; panelcn.MOPS 21,40; VisCap 147,90.

GATK-gCVN execution excluding the optional explicit GC-content-based filtering step

We followed the steps indicated in the GATK guide to call rare germline variants. In this guide, section 2, which includes AnnotateIntervals and FilterIntervals steps, is optional. Moreover, the AnnotateIntervals step, which involves GC-content-based filtering, is also described as optional within section 2. We hence decided to compare three workflows:

- GATK-gCNV. It is the final GATK-gCNV workflow used in all benchmark sections and includes AnnotateIntervals and FilterIntervals steps.
- GATK-gCNV_no_AI. It excludes the AnnotateInterval step, which is the default approach in the GATK WDL germline cohort workflow.
- GATK-gCNV_no_AI_FI. It excludes both optional AnnotateIntervals and FilterIntervals steps.

The following tables and figures show a performance comparison of three GATK-gCNV workflows.

Performance at ROI level

dataset	algorithm	TP	TN	FP	FN	total	sensitivity	specificity	F1
ICR96	GATK-gCNV	292	28382	128	4	28806	0.9865	0.9955	0.8156
	GATK-gCNV_no_AI	289	28381	129	7	28806	0.9764	0.9955	0.8095
	GATK-gCNV_no_AI_FI	287	28414	96	9	28806	0.9696	0.9966	0.8454
panelcnDataset	GATK-gCNV	315	9476	10	6	9807	0.9813	0.9989	0.9752
	GATK-gCNV_no_AI	315	9476	10	6	9807	0.9813	0.9989	0.9752
	GATK-gCNV_no_AI_FI	303	9482	4	18	9807	0.9439	0.9996	0.9650
inHouseMiSeq	GATK-gCNV	497	4128	116	29	4770	0.9449	0.9727	0.8727
	GATK-gCNV_no_AI	496	4165	79	30	4770	0.943	0.9814	0.901
	GATK-gCNV_no_AI_FI	452	4181	63	74	4770	0.8593	0.9852	0.8684
inHouseHiSeq	GATK-gCNV	381	4123	135	14	4653	0.9646	0.9683	0.8364
	GATK-gCNV_no_AI	386	4122	136	9	4653	0.9772	0.9681	0.8419
	GATK-gCNV_no_AI_FI	352	4184	74	43	4653	0.8911	0.9826	0.8575

Performance at gene level

dataset	algorithm	TP	TN	FP	FN	total	sensitivity	specificity	F1
ICR96	GATK-gCNV	67	1736	16	1	1820	0.9853	0.9909	0.8874
	GATK-gCNV_no_AI	66	1734	18	2	1820	0.9706	0.9897	0.8684
	GATK-gCNV_no_AI_FI	64	1735	17	4	1820	0.9412	0.9903	0.8591
panelcnDataset	GATK-gCNV	41	414	2	0	457	1.0000	0.9952	0.9762
	GATK-gCNV_no_AI	41	414	2	0	457	1.0000	0.9952	0.9762
	GATK-gCNV_no_AI_FI	33	416	0	8	457	0.8049	1.0000	0.8919
inHouseMiSeq	GATK-gCNV	61	170	2	3	236	0.9531	0.9884	0.9606
	GATK-gCNV_no_AI	60	168	4	4	236	0.9375	0.9767	0.9375
	GATK-gCNV_no_AI_FI	42	164	8	22	236	0.6562	0.9535	0.7368
inHouseHiSeq	GATK-gCNV	55	171	7	3	236	0.9483	0.9607	0.9167
	GATK-gCNV_no_AI	56	171	7	2	236	0.9655	0.9607	0.9256
	GATK-gCNV_noAI_FI	41	171	7	17	236	0.7069	0.9607	0.7736

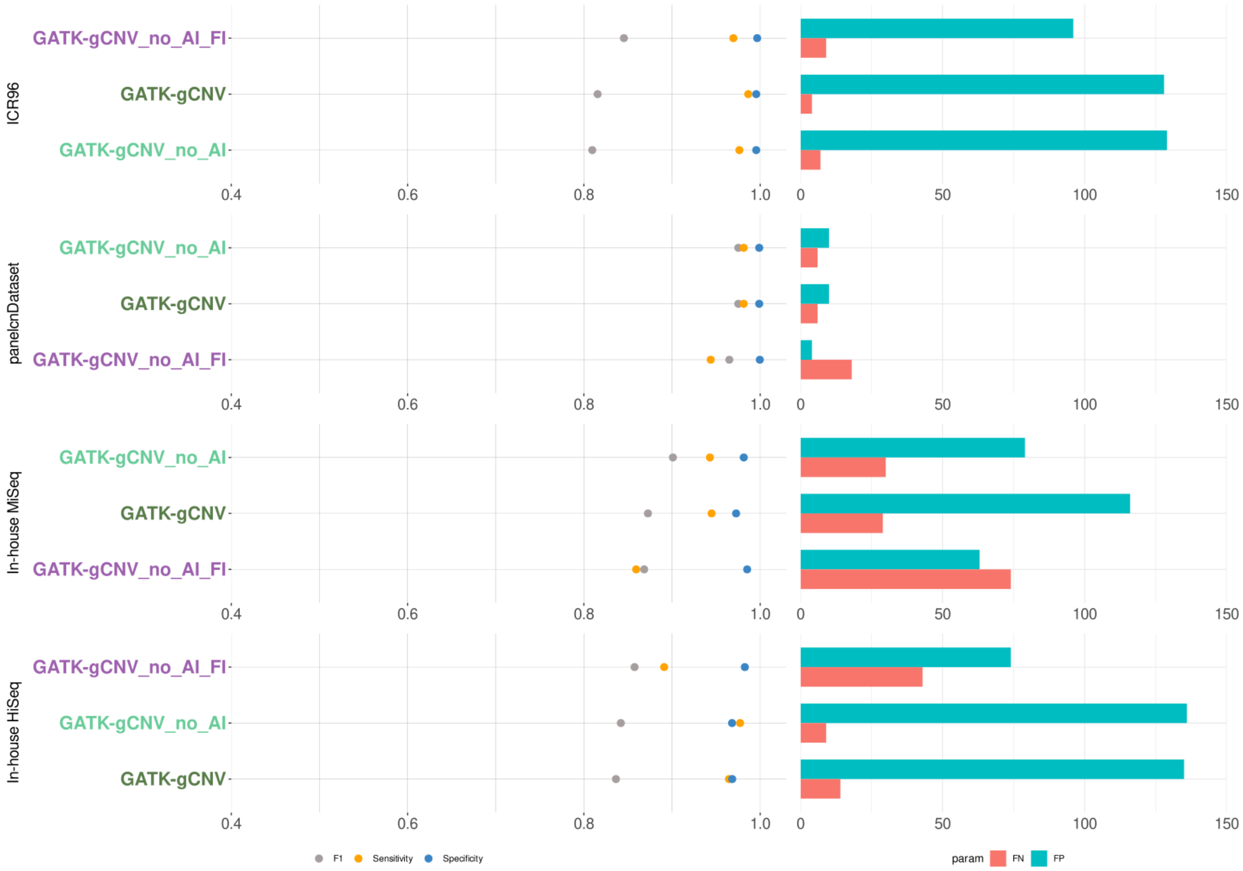

Comparison of three GATK-gCNV workflows at ROI level. GATK-gCNV is the final GATK-gCNV workflow used in all benchmark sections and includes AnnotateIntervals and FilterIntervals steps. GATK-gCNV_no_AI excludes the AnnotateInterval step, which is the default approach in the GATK WDL germline cohort workflow. GATK-gCNV_no_AI_FI excludes AnnotateIntervals and FilterIntervals steps. Results are ranked according to their F1 scores in each dataset. (FN false negative; FP false positive; F1 F1 score).

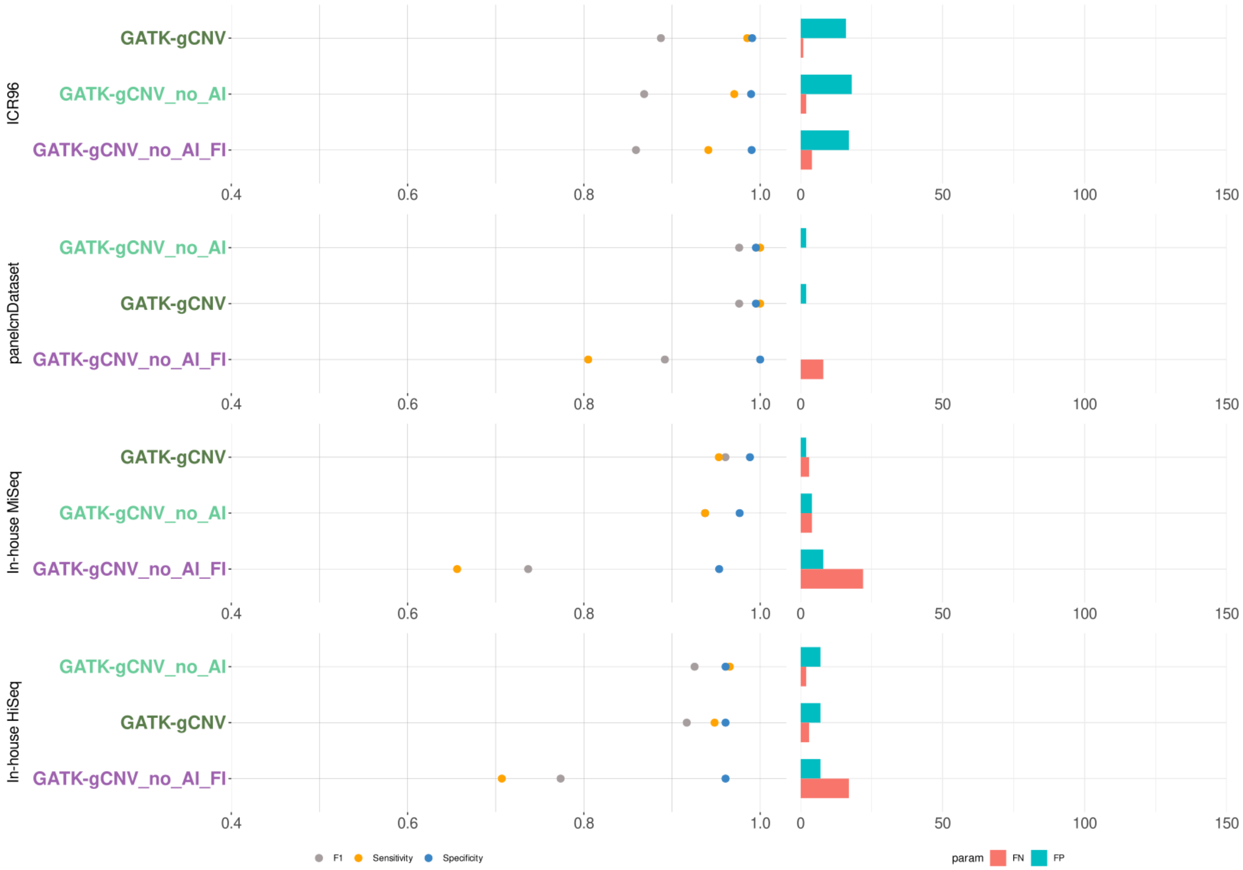

Comparison of three GATK-gCNV workflows at gene level. GATK-gCNV is the final GATK-gCNV workflow used in all benchmark sections and includes AnnotateIntervals and FilterIntervals steps. GATK-gCNV_no_AI excludes the AnnotateInterval step, which is the default approach in the GATK WDL germline cohort workflow. GATK-gCNV_no_AI_FI excludes AnnotateIntervals and FilterIntervals steps. Results are ranked according to their F1 scores in each dataset. (FN false negative; FP false positive; F1 F1 score).

Tools achieving sensitivity 1 across all datasets at the gene level

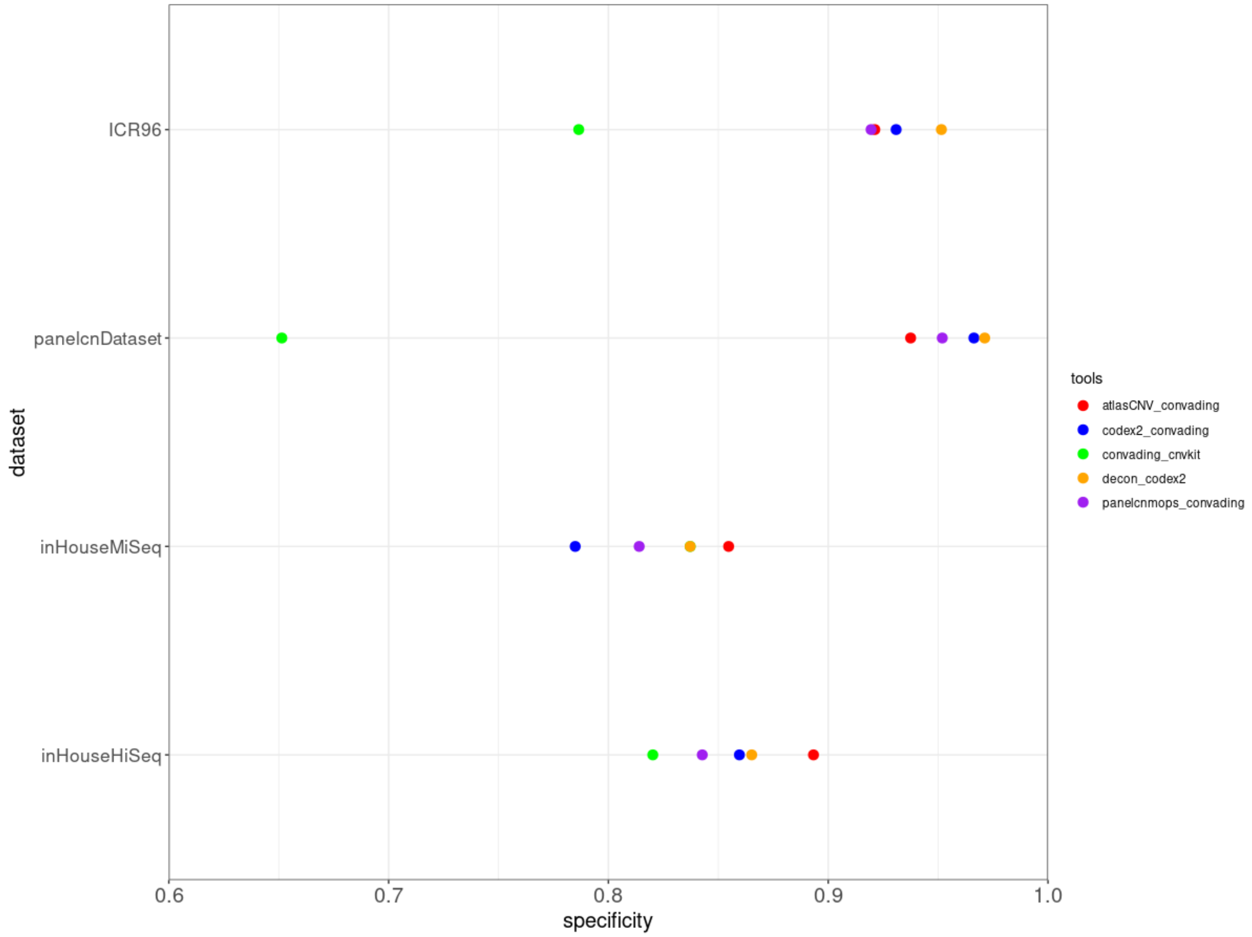

Specificity of tool pairs achieving perfect sensitivity across all datasets at the gene level. In the InhouseMiSeq dataset, convading_cnvkit achieved the same specificity as decon_codex2, hence only 4 points are shown.
