# Supplementary figures and images for: Detection of germline CNVs from gene panel data: benchmarking the state of the art
Source: Brief Bioinform. 2024 Dec 12;26(1):bbae645. doi: 10.1093/bib/bbae645 (PMC11637760; doi:10.1093/bib/bbae645)

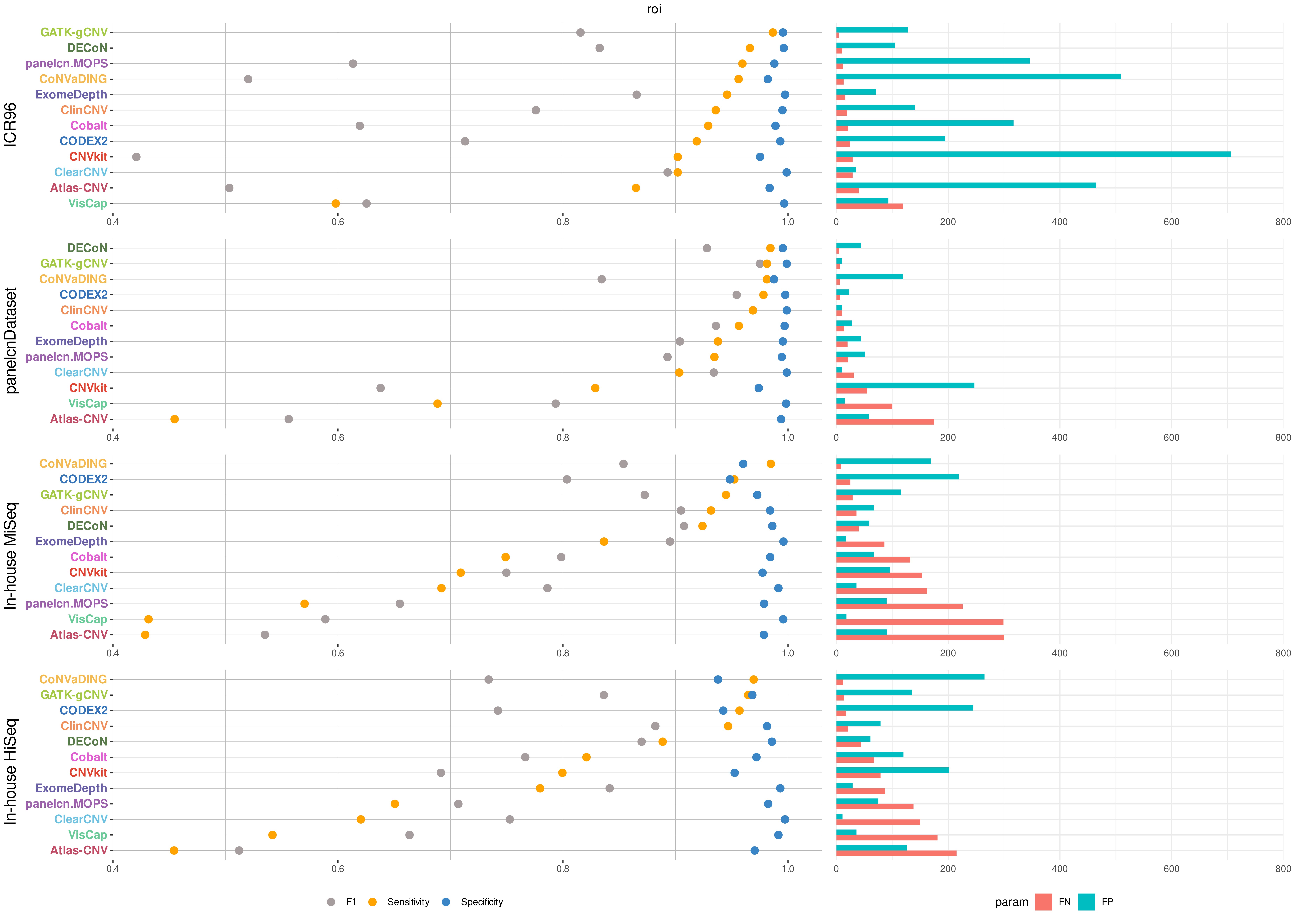

Supplement: Supplementary_data_bbae645 [file supplementary_data_bbae645.zip › Supplementary Figure 1.jpg]

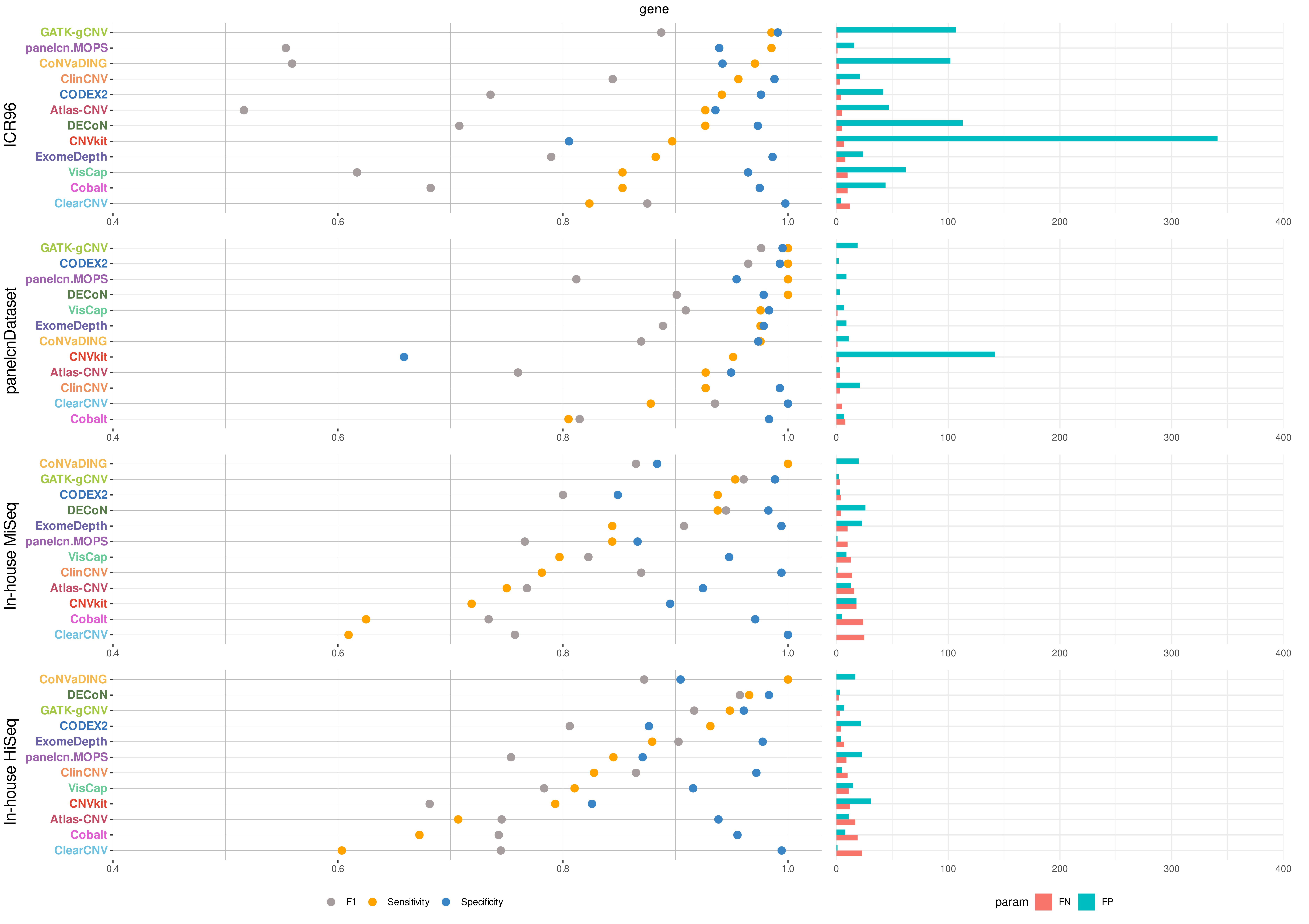

Supplement: Supplementary_data_bbae645 [file supplementary_data_bbae645.zip › Supplementary Figure 2.jpg]
